# A Potential Bidirectional Interaction Between Heart Failure and Cancer Progression: Insights From Two Rapidly Fatal Cases

**DOI:** 10.7759/cureus.116668

**Published:** 2026-09-21

**Authors:** Mohamed Djerouni, Ramy Abbas, Soufiane Khelifi Touhami, Omar Alotaibi, Fouzia Kauser, Wael Abdelmottaleb, Abdulrahman Alrubayee

**Affiliations:** 1 Medical Oncology, King Abdulaziz Specialist Hospital, Taif, SAU; 2 Medical Oncology, King Abdulaziz Specialist Hospital, TAIF, SAU; 3 Radiology, King Abdulaziz Specialist Hospital, Taif, SAU; 4 Pathology, King Abdulaziz Specialist Hospital, Taif, SAU; 5 Internal Medicine, New York Medical College / Metropolitan Hospital, New York, USA; 6 Oncology, King Abdulaziz Specialist Hospital, Taif, SAU

**Keywords:** cancer progression, cardiomyopathy, cardio-oncology, heart-cancer axis, heart failure

## Abstract

The interaction between malignancy and cardiovascular disease is increasingly recognized in cardio-oncology. While cancer therapy-related cardiac dysfunction is well established, a potential bidirectional relationship between heart failure (HF) and cancer progression remains underexplored. We present the cases of two patients without known cardiovascular disease who developed new-onset HF in temporal association with rapidly progressive metastatic malignancies. A 65-year-old man with metastatic pancreatic adenocarcinoma developed severe left ventricular (LV) systolic dysfunction with a left ventricular ejection fraction (LVEF) of 30%, apical akinesis, and suspected LV thrombus after a previously normal cardiac evaluation. A 44-year-old man with metastatic esophageal squamous cell carcinoma developed severe LV systolic dysfunction (LVEF 25-30%) with global hypokinesia, also following a normal baseline cardiac assessment. In both cases, baseline electrocardiography, troponin levels, and echocardiography were normal, whereas B-type natriuretic peptide (BNP) levels became elevated during rapid tumor progression. Both patients experienced rapid clinical deterioration and died within weeks of diagnosis. These cases highlight a potential interaction between aggressive malignancy and cardiac dysfunction, possibly mediated by inflammatory, metabolic, and neurohormonal mechanisms. Conversely, HF may adversely affect oncologic outcomes by reducing physiological reserve and limiting therapeutic options. Although causality cannot be established from these observations, new-onset HF occurring alongside rapidly progressive malignancy may represent an underrecognized clinical phenomenon with important prognostic implications.

## Introduction

Cardio-oncology has emerged as a multidisciplinary field addressing the cardiovascular complications of cancer and its therapies [1]. While cancer therapy-related cardiac dysfunction (CTRCD) is well characterized, increasing evidence suggests a more complex and potentially bidirectional relationship between malignancy and cardiovascular disease [2,3]. Experimental and clinical data indicate that heart failure (HF) and cancer share common pathophysiological pathways, including chronic inflammation, neurohormonal activation, metabolic dysregulation, and immune dysfunction [2,3].

However, clinical evidence demonstrating a temporal and potentially bidirectional interaction remains limited. We present two cases of rapidly progressive metastatic malignancy associated with new-onset HF, highlighting a possible reciprocal relationship.

## Case presentation

Case 1

A 65-year-old previously healthy man presented with abdominal pain and weight loss. Computed tomography revealed a 3 × 3 cm pancreatic head mass with innumerable bilobar hepatic metastases, with the largest measuring 3.7 × 2.8 cm (Figure 1). Liver biopsy confirmed metastatic adenocarcinoma of pancreaticobiliary origin with a Ki-67 index of 65%. With preserved performance status, palliative chemotherapy was planned. Baseline cardiac assessment showed no evidence of cardiac dysfunction, and echocardiography demonstrated normal cardiac structure and function with a left ventricular ejection fraction (LVEF) of 67%.

**Figure 1:**
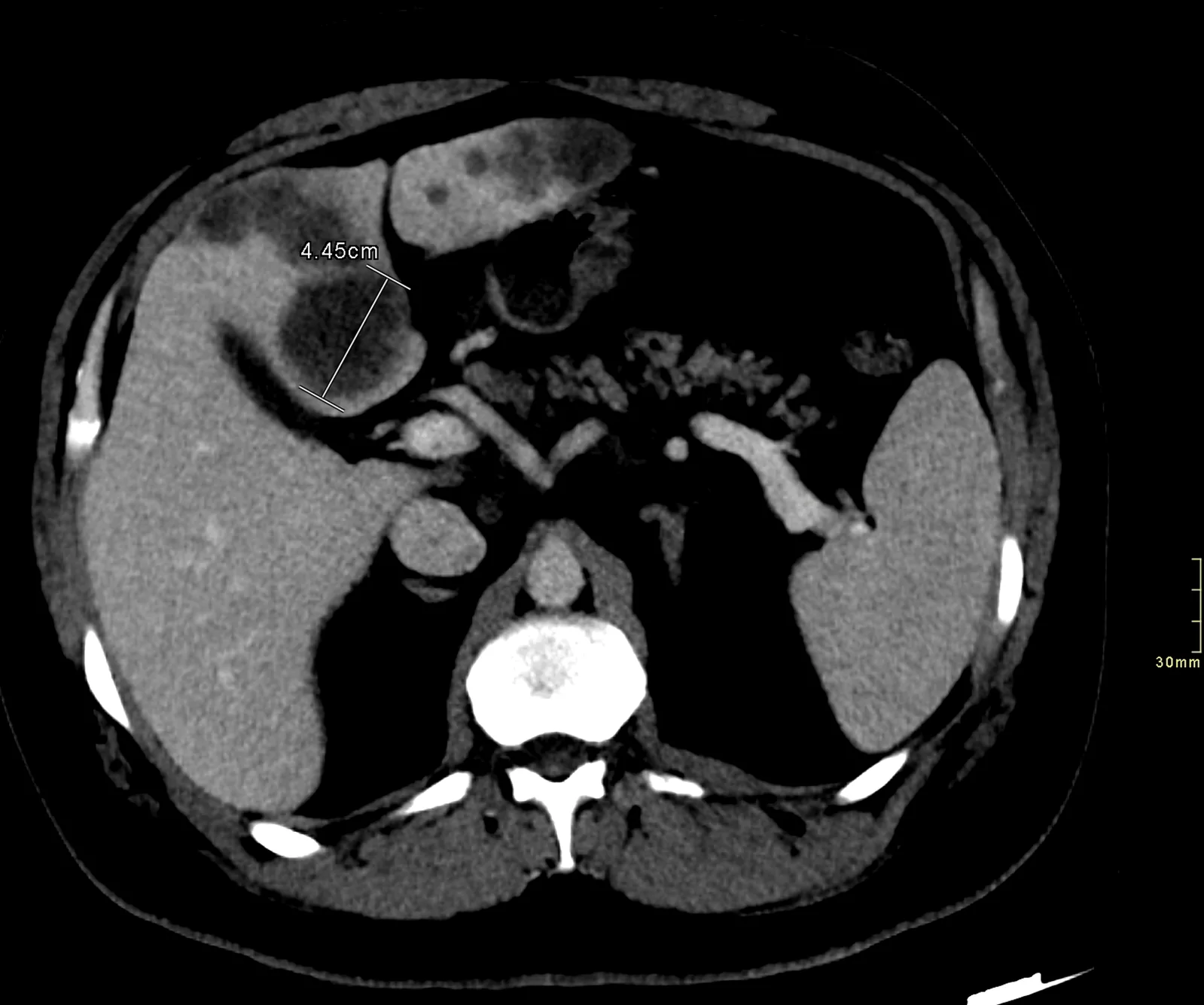
Baseline CT scan showing liver metastases

Within weeks, before initiation of chemotherapy, he presented again with jaundice, clinical deterioration, and rising bilirubin levels. Magnetic resonance cholangiopancreatography demonstrated rapid tumor progression, with enlargement of the hepatic metastases, with the largest measuring 7.2 × 4.5 cm, worsening biliary dilatation, and new ascites (Figure 2). Repeat echocardiography demonstrated new severe left ventricular (LV) systolic dysfunction with an LVEF of 30%, apical akinesis, and laminated apical thrombus, with mild mitral regurgitation and no pericardial effusion (Figure 3). B-type natriuretic peptide (BNP) was elevated during this period of rapid disease progression. Despite supportive management, the patient developed progressive multiorgan failure and died within weeks.

**Figure 2:**
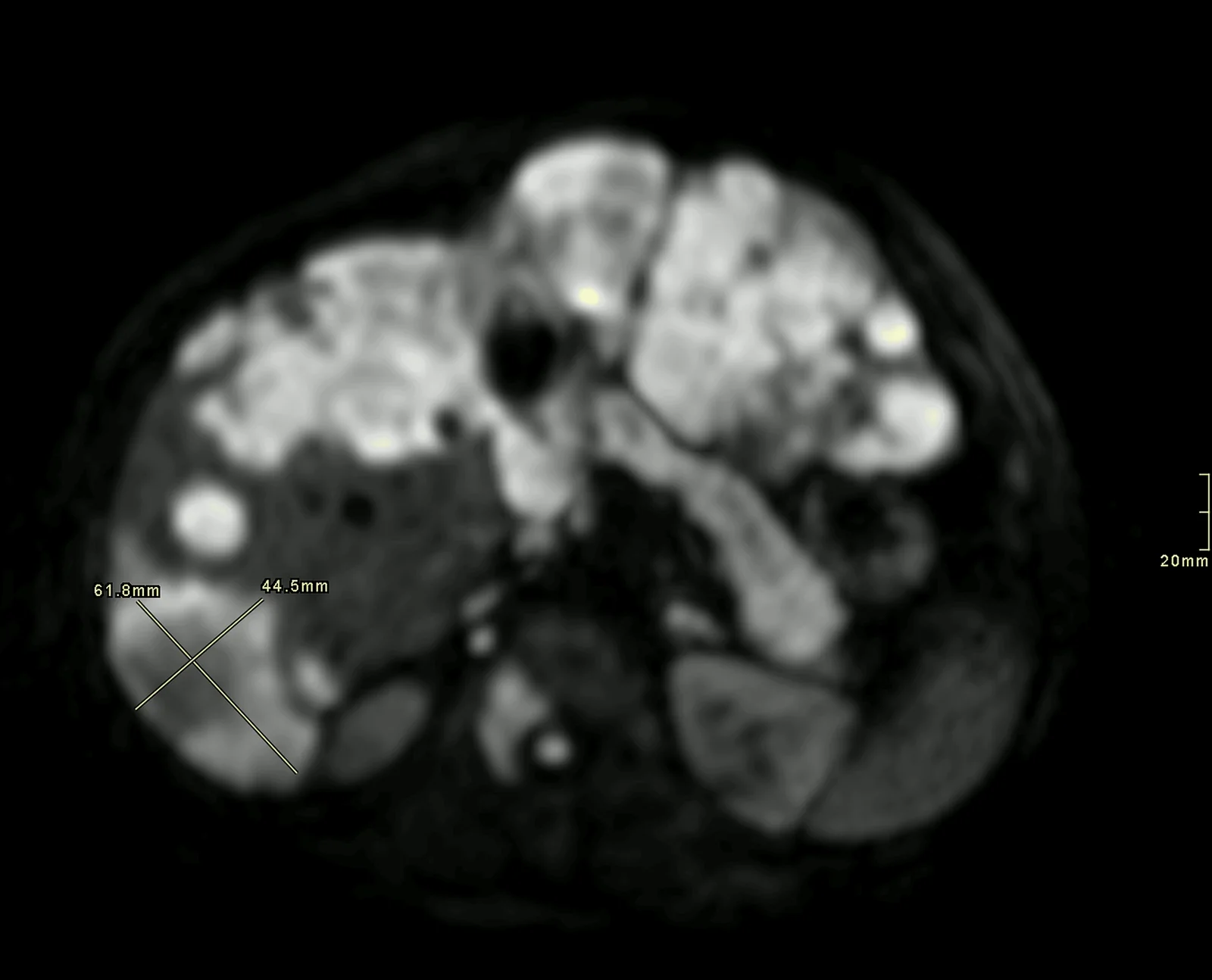
MRI performed following clinical deterioration showing radiological progression of liver metastases

**Figure 3:**
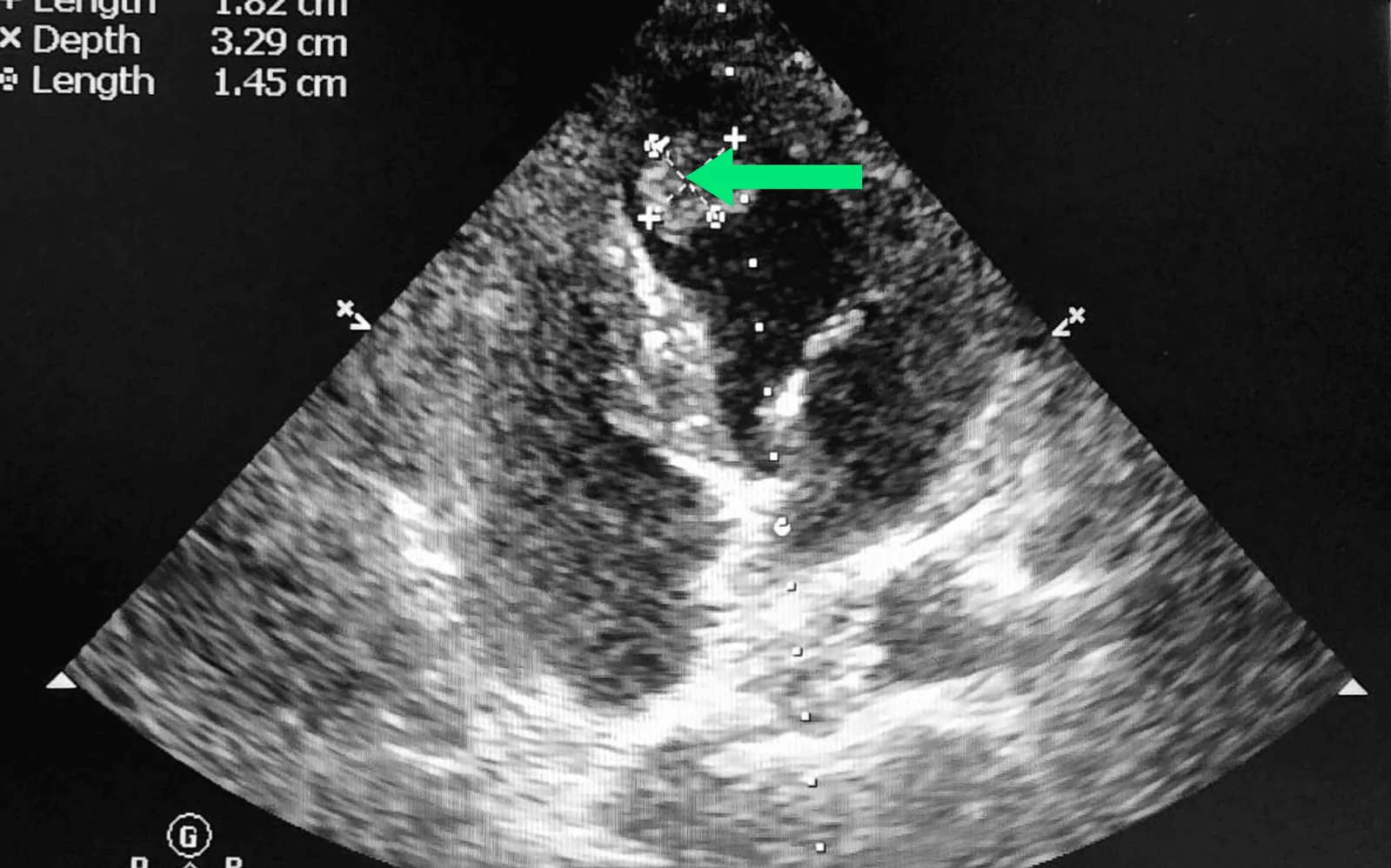
Echocardiogram showing laminated apical thrombus (green arrow)

Case 2

A 44-year-old previously healthy heavy smoker presented with abdominal pain and dysphagia. Endoscopic biopsy confirmed poorly differentiated invasive esophageal squamous cell carcinoma with a Ki-67 index of 70%. Computed tomography demonstrated a gastroesophageal junction mass with bilobar hepatic metastases, with the largest measuring 4.2 cm, a right adrenal metastasis, and extensive lymphadenopathy (Figure 4). His performance status was ECOG 1.

**Figure 4:**
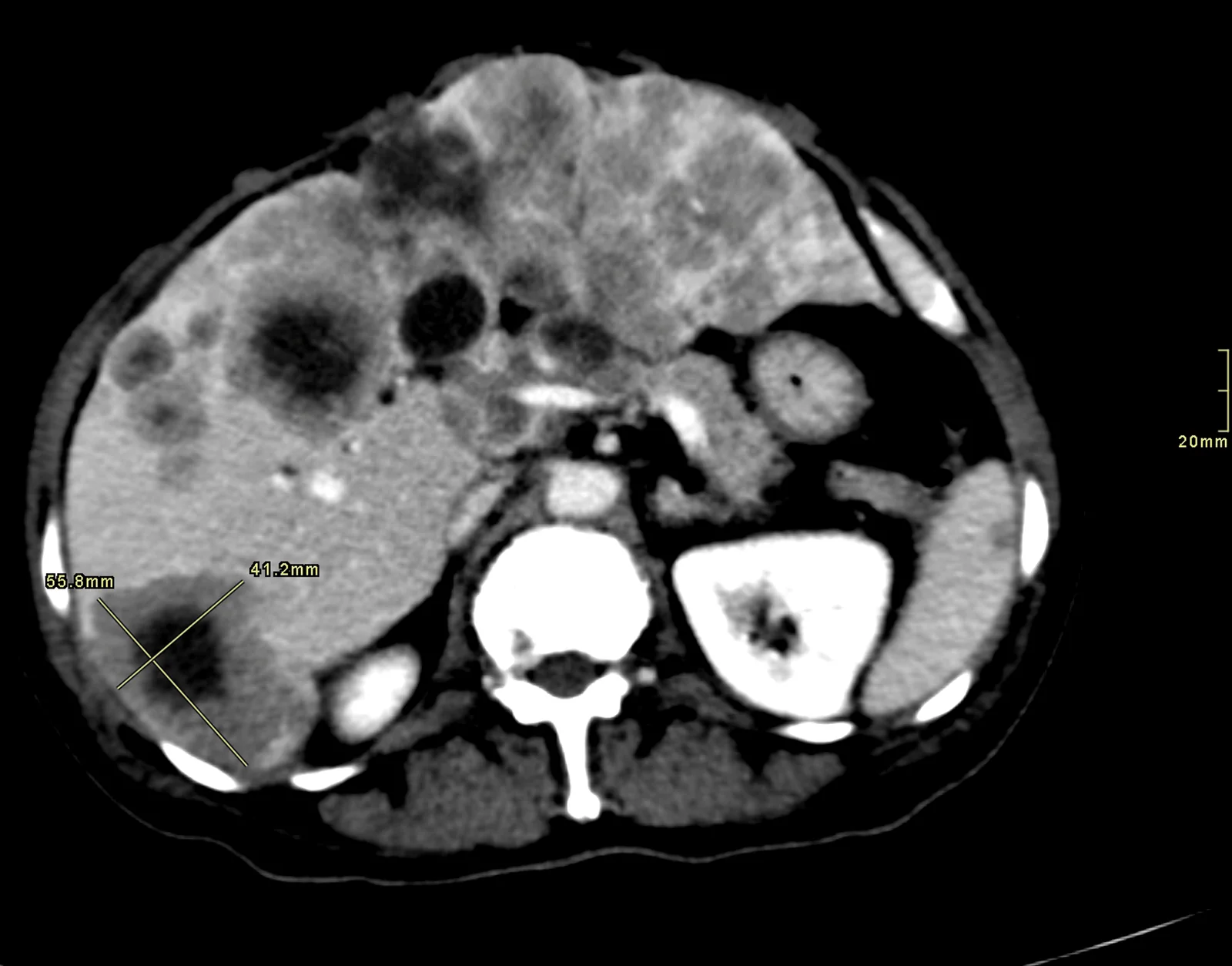
Baseline CT scan showing enlarged bilobar liver metastasis

During hospitalization, he developed progressive hyperbilirubinemia and new-onset dyspnea. Echocardiography revealed a dilated LV with severe systolic dysfunction (LVEF 25-30%), global hypokinesia, mild mitral valve prolapse, and no pericardial effusion (Figure 5). BNP was elevated. Subsequent imaging demonstrated marked tumor progression, with enlargement of hepatic metastases up to 7.4 cm and new biliary dilatation (Figure 6). His condition rapidly deteriorated, culminating in multiorgan failure and death within weeks.

**Figure 5:**
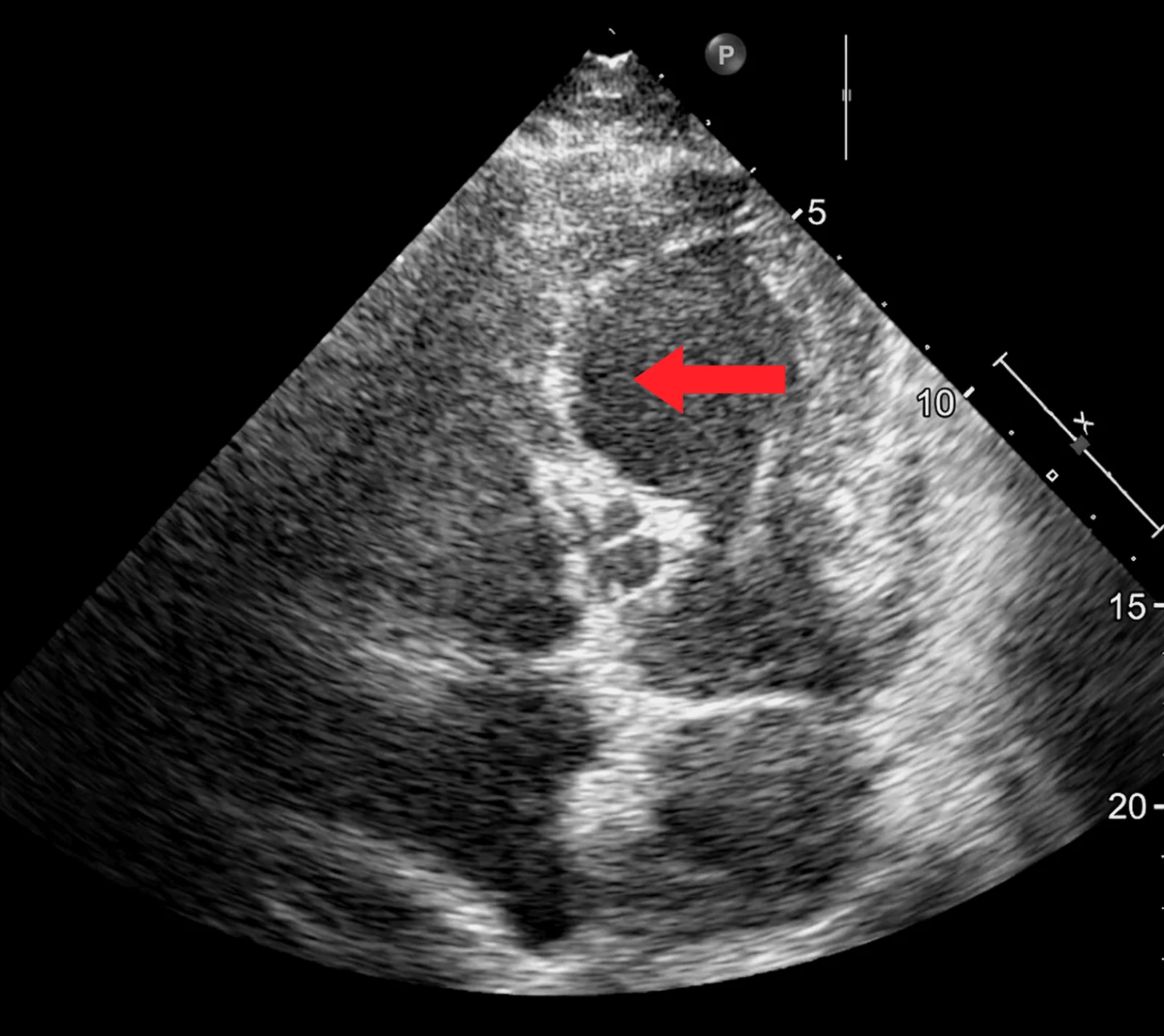
Echocardiogram showing severe systolic dysfunction The red arrow indicates an enlarged left ventricular cavity, demonstrating left ventricular dilatation

**Figure 6:**
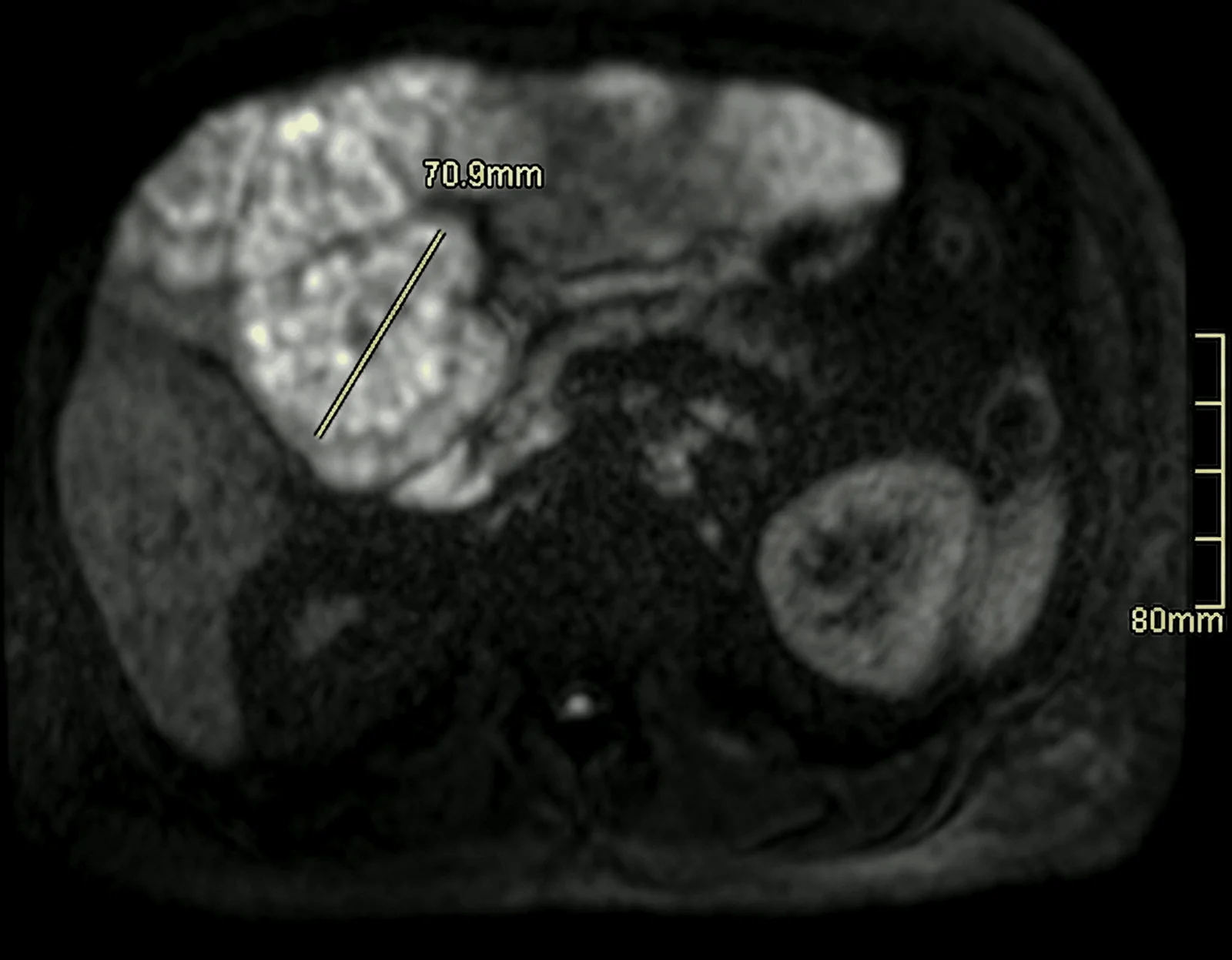
MRI performed two weeks after the baseline CT scan showing rapid progression of extensive liver metastases in size and number

## Discussion

These cases demonstrate a temporal association between rapid cancer progression and the development of HF in patients without previously documented cardiovascular disease. Both patients had preserved baseline cardiac function and subsequently developed severe LV systolic dysfunction before initiation of systemic anticancer therapy. This temporal pattern raises the possibility of malignancy-associated cardiac dysfunction independent of conventional cancer therapy-related cardiotoxicity. Potential mechanisms include systemic inflammation, metabolic and cachectic stress, neurohormonal activation, and tumor-related circulating factors [2,3]. In addition, the suspected LV thrombus in case 1 may reflect the interaction between severe LV dysfunction and the prothrombotic state associated with advanced malignancy.

The relationship between HF and cancer is increasingly recognized as potentially bidirectional and multifactorial. Experimental studies provide evidence that cardiac dysfunction may influence tumor biology. Meijers et al. reported that HF stimulated tumor growth through circulating factors and identified several cardiac-derived proteins, including SerpinA3, associated with tumor-promoting effects [4]. Subsequently, Avraham et al. demonstrated that pressure overload-induced cardiac remodeling accelerated primary tumor growth and metastatic spread in experimental models. Serum obtained from animals with cardiac remodeling also enhanced cancer cell proliferation in vitro, supporting the presence of cardiac-derived circulating tumor-promoting factors [5].

Beyond these experimental observations, HF and cancer share several biological pathways, including chronic inflammation, oxidative and metabolic stress, sympathetic nervous system activation, renin-angiotensin-aldosterone system activation, and clonal hematopoiesis of indeterminate potential [2,3]. Emerging evidence suggests that the failing heart may influence cancer progression through secreted factors, or cardiokines, as well as neurohormonal activation, systemic inflammation, and epigenetic remodeling of bone marrow-derived cells [3]. Together, these observations have contributed to the emerging concept of a “heart-cancer axis.”

The reciprocal direction of this relationship is also increasingly recognized. Cancer itself, independent of anticancer therapy, may adversely affect cardiovascular function. Proposed mechanisms include systemic inflammatory activation, cachexia-related cardiac wasting, metabolic remodeling, tumor-derived factors, and cancer-derived extracellular vesicles capable of influencing myocardial structure and function [3]. These mechanisms provide biological plausibility for cardiac dysfunction occurring during aggressive malignancy even in the absence of exposure to recognized cardiotoxic therapies.

In both cases, severe LV systolic dysfunction developed over a short period contemporaneously with marked tumor progression. Importantly, neither patient had received systemic anticancer therapy before the onset of HF, making conventional CTRCD an unlikely explanation. The high proliferative indices and rapidly increasing metastatic burden observed in both cases further underscore the aggressive biological behavior of the malignancies. However, these findings alone cannot establish a direct mechanistic relationship between tumor progression and cardiac dysfunction.

Alternative explanations for the observed LV dysfunction must also be considered. These include occult ischemic heart disease, stress cardiomyopathy, myocarditis, and other causes of nonischemic cardiomyopathy. In case 1, the presence of apical akinesis and suspected LV thrombus makes an ischemic or stress-related mechanism particularly relevant to the differential diagnosis. In case 2, global hypokinesia and LV dilatation are compatible with several potential etiologies. Because comprehensive etiological investigations such as coronary angiography, cardiac magnetic resonance imaging, or endomyocardial biopsy were not performed, a causal relationship between malignancy and HF cannot be established. Accordingly, these observations should be regarded as hypothesis-generating.

Nevertheless, the close temporal association between marked tumor progression and severe cardiac dysfunction in two patients without prior documented cardiac disease or exposure to systemic anticancer therapy is clinically noteworthy. These cases support the need for further investigation of the potential heart-cancer axis in patients with aggressive malignancies.

## Conclusions

In the two reported patients, new-onset HF occurred during a period of rapidly progressive malignancy in the absence of previously documented cardiovascular disease or exposure to systemic anticancer therapy. Severe LV systolic dysfunction developed contemporaneously with rapid metastatic progression and was followed by clinical deterioration, multiorgan failure, and death within weeks. Neither patient was able to proceed with the planned systemic anticancer therapy because of the rapid deterioration in clinical condition.

Although the temporal association is noteworthy, these observations do not establish a causal relationship between cancer progression and cardiac dysfunction, and alternative cardiovascular etiologies cannot be excluded. Nevertheless, the findings are compatible with emerging experimental and clinical observations suggesting potential biological interactions between cancer and HF. Further prospective and mechanistic studies are needed to clarify whether malignancy itself may contribute to cardiac dysfunction in selected patients and to better define the potential clinical relevance of the proposed heart-cancer axis.
